# Development of an arm support system to improve ergonomics in laparoscopic surgery: study design and provisional results

**DOI:** 10.1007/s00464-014-3984-x

**Published:** 2014-12-25

**Authors:** Benjamin Steinhilber, Sascha Hoffmann, Kristian Karlovic, Stefan Pfeffer, Thomas Maier, Omar Hallasheh, Stephan Kruck, Robert Seibt, Monika A. Rieger, Michael Heidingsfeld, Ronny Feuer, Oliver Sawodny, Ralf Rothmund, Karl-Dietrich Sievert

**Affiliations:** 1 Institute of Occupational and Social Medicine and Health Services Research, University Hospital Tübingen, Tübingen, Germany; 2 Department of Gynecology and Obstetrics, University Hospital Tübingen, Tübingen, Germany; 3 Institute for Engineering Design and Industrial Design (IKTD), University of Stuttgart, Stuttgart, Germany; 4 Department of Urology, University Hospital Tübingen, Tübingen, Germany; 5 Institute for System Dynamics (ISYS), University of Stuttgart, Stuttgart, Germany; 6 Department of Urology, University Hospital Lübeck, Schleswig-Holstein, Lübeck, Germany

**Keywords:** Laparoscopic surgery, Stress, Strain, Ergonomic, Human/robotic, Urology, Gynaecology & obstetrics

## Abstract

**Background:**

Laparoscopic surgery (LS) induces physical stress to the surgeon that is associated with an increased prevalence of musculoskeletal pain and injury in the shoulder–neck region. The aim of this research project is to develop an arm support system (ASsyst) that reduces physical stress and is applicable to various laparoscopic interventions and operation room settings.

**Methods:**

A systematic approach to develop an ASsyst started in October 2012 consisting of five consecutive steps. In step 1, 14 laparoscopic interventions were observed using subjective and objective measures to determine key indicators for the conception of an ASsyst in LS. In step 2, an expert workshop was held to find and evaluate solutions to generate concepts for a support system based on the results of step 1 and general methods. During the third step, prototypes of ASsyst were tested in an experimental setting. Steps 4 and 5 are currently in process and include the final development of the ASsyst using the most promising concept for the evaluation during simulated LS.

**Results:**

Increased levels of physical stress were found in LS. Asymmetric strains were common. Three prototypes of ASsyst emerged from step 1 and 2. These prototypes were a cable construction with a noose for the lower arm, a support from below the elbow and a pneumatic vest supporting the upper arm. The experimental testing of these prototypes demonstrated reduced physical stress when compared to the unsupported environment. The support from below the elbow seemed to be the most practical in terms of implementation in various operation room settings and acceptance by surgeons. Step 4 and 5 are still in process.

**Conclusions:**

Ergonomic problems have been identified in LS that could be addressed by an ASsyst. The concept of supporting the elbow from below has been found to be the most promising approach.

Laparoscopic surgery (LS) is a common surgical method that provides several advantages to the patient. It is associated with reduced trauma, faster recovery, and reduced rate of failed septic wound healing, when compared to open surgery [1, 2]. However, the biomechanical and mental strains placed on a surgeon when performing laparoscopic procedures are significantly higher, resulting in increased muscle fatigue [3], musculoskeletal pain [4], and injury [5]. In particular, the shoulder and neck region as well as the upper limbs are reported to be affected [6, 7]. Further confounding these statistics are the fact that workload intensity among surgeons [8] may escalate its adverse aspects and may even increase the risk of medical error.

Innovative technical developments such as robotic-assisted laparoscopy appear to provide an opportunity to reduce physical stress on surgeons [9]. However, robotic-assisted laparoscopy aims to further improve patient outcomes [10], not necessarily to improve ergonomics. It is therefore not surprising that muscle strain in the shoulder–neck region continues to be reported, despite this new technology [11].

The aim of this research project is to develop an arm support system (ASsyst) that addresses the ergonomic issues in LS to reduce the amount of physical stress in the shoulder and neck region, as well as the upper limbs of the surgeon. This paper provides an overview of the steps in the development of ASsyst for LS and summarizes the major results of each step.

## Materials and methods

Three university institutes, two clinical departments and two industrial partners participated in this project. Table 1 provides an overview of the five project steps along with the corresponding aim, method, and responsible project partners.

**Table 1:** Consecutive steps of the research project

Step	Aim	Methods	Responsible project partner
1	Investigate current situation	• Cross-sectional study on laparoscopic interventions	• Institute of Occupational and Social Medicine and Health Services Research
			• Institute for Engineering Design and Industrial Design
			• Department of Gynecology and Obstetrics
			• Department of Urology
2	Generate ideas for development of a support system	• Expert workshop	• All university institutes and departments
			• All industrial partners
3	Test and evaluate elementary concepts/prototypes	• Experimental design with data acquisition during simulated laparoscopic interventions	• Institute of Occupational and Social Medicine and Health Services Research
			• Institute for Engineering Design and Industrial Design
4	Develop arm support system	• Hardware construction and software development	• Institute of Engineering Design and Industrial Design
			• Institute for System Dynamics
			• Industrial partners
5	Evaluate arm support system	• Cross-sectional study on simulated laparoscopic interventions with and without an arm support system	• Institute of Occupational and Social Medicine and Health Services Research
			• Institute for Engineering Design and Industrial Design
			• Department of Gynecology and Obstetrics
			• Department of Urology

### Step 1: investigate current situation

Data acquisition took place over 12 months at two surgical units (Urology and Gynecology) by analyzing surgeons and their operating procedures during LS as well as the operating room setup [12].

Fourteen standard laparoscopic surgical interventions were identified and observed in the urological clinic (nephrectomy, and partial nephrectomies; *n* = 4) and the gynecological clinic (hysterectomy, ovariectomy; *n* = 10). Laparoscopic procedures were similar within each of the two surgical units and were accomplished by six experienced surgeons (work experience >10 years, five right-handed and one left-handed males with four gynecological and two urological surgeons). In order to consider various sources of physical stress and physical complaints, a multiple measurement approach consisting of subjective and objective methods was used. Subjective methods included the NASA TLX [13] and the Nordic Questionnaire [14]. The NASA TLX questionnaire [13] determines the workload during a specific work task according to its six dimensions: physical demand, mental demand, temporal demand, performance, effort and frustration. This questionnaire provides an overall weighted workload score (OWWS) including all of the aforementioned dimensions and more detailed scores of every dimension. Higher scores indicate higher demands. The Nordic Questionnaire [14], a standard tool to assess prevalence of musculoskeletal disorders in specific regions of the body, was used to obtain information about musculoskeletal complaints of the last week and last 12 months. The Nordic Questionnaire was completed prior to the first LS and after every LS, the surgeon completed a NASA TLX questionnaire.

The objective methods included: a posture sensor placed on the dominant arm, bipolar surface electromyograms (SEMG) located on the right and left trapezius muscle (pars descendens), heart rate, and 2-dimensional (2D) video analysis.

#### Posture sensor

A three-dimensional gravimetric posture sensor (resolution: 0.1° angle and 125 ms in time; maximum static error: 0.5°, THUMEDI, Thum-Jahnsbach, Germany) was placed at the lateral part of the dominant upper arm. This sensor measures the inclination toward the perpendicular line. In the case of an upright torso posture, as applied in this study, it is possible to measure the arm abduction angle as the inclination in the frontal plane.

SEMGs were measured at the trapezius pars descendens muscles of the dominant arm. The skin was prepared by cleansing with abrasive paste (Nurpreb^®^) and shaving if there was excessive body hair. Self-sticking silver/silver chloride (Ag/AgCl) electrodes with an active diameter of 15 mm and an inter-electrode distance of 25 mm were used. Measurements were conducted with a PS11 measurement device (THUMEDI, Thum-Jahnsbach, Germany). The SEMG raw signal was sampled with 2048 Hz, digitalized, and low and high-pass filtered (12, 650 Hz, 11th order). After the Fast Fourier Transformation (1024 FFT-points, Bartlett-Window with 50 % overlap), the electrical activity (EA) was calculated as the root mean square of the amplitude from the power spectrum. SEMGs were normalized to a submaximal static reference contraction with an ante-version of both straight arms holding a dumbbell (2 kg) in each hand [12].

#### Videoanalysis

**Fig. 1:**
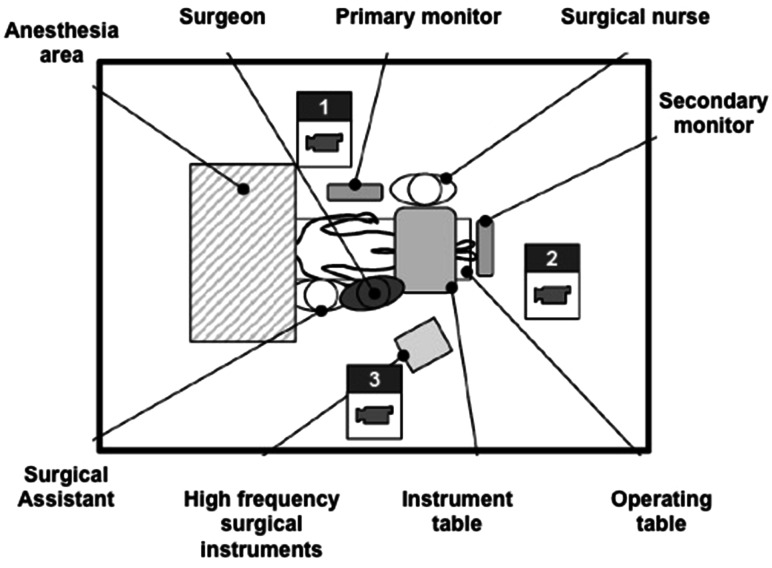
Setup of the video analysis measurements in the operating room

The 2D video analysis was performed using three video cameras at different angles (back, front and side view) in order to observe the entire body of the surgeon and to obtain information about movements, postures, and the individual surgeons’ procedures (Fig. 1). Two experts in ergonomy independently screened the recorded videos for extreme postures/movements and frequent unergonomic postures/movements. Significant postures based on expert ratings were then depicted using Dartfish-software (Fribourg, Switzerland).

Measures (arm posture, SEMG, and 2D video analysis) were recorded continuously throughout the laparoscopic interventions.

### Step 2: generate ideas for development of a support system

An expert workshop, including all project partners, was conducted to generate a basic conceptual design of a support system. Due to the cooperative approach of this project, three of the investigated surgeons and the four evaluators who applied the measurements during step 1, also participated in this workshop. During the initial expert workshop, the results of step 1 were presented and extensively discussed. The surgeons and evaluators contributed important information from the ‘field’ location. Concepts were then generated using different well-established engineering methods [15].

### Step 3: test and evaluate elementary concepts/prototypes

Three rudimentary prototypes were constructed and tested in an experimental setting by applying the same objective measures (posture sensor, SEMG, heart rate, and 2D video analysis) used during step 1. One gynecological surgeon performed the laparoscopic simulation exercise. The demonstration surgery was first performed without the support system and subsequently using each of the three prototypes. The simulation exercise was carried out using a pelvi-trainer and standard laparoscopic instruments, i.e., a part of the educational program of the European Society for Gynaecological Endoscopy (ESGE) the ‘Laparoscopic Skills Testing and Training model (LASTT).’ The setup included the standing position of the surgeon, monitor position, and height, as well as working height. The intention was to simulate the operating setup observed under real conditions in the gynecological clinic.

### Step 4 and 5: development and evaluation of the prototype

Steps 1 through 3 yielded one prototype that supports the elbow from below. Using the same surgeons, step 5 of the project involves evaluating the system using the same multiple measurement approach in step 1.

## Results

### Step 1: investigate current situation

The results of the Nordic Questionnaire showed that surgeons from gynecology primarily indicated stress problems in the shoulder region, neck, and lower back region. Three subjects reported complaints in the neck region on 1–7 and 8–30 days within the last year and one subject complained about problems in the shoulder region that lasted up to more than 30 days within the last year. In urology data from the Nordic Questionnaire was not available. However, work ability of these two surgeons were not impaired. The OWWS of the NASA TLX was comparable between the two surgical units. The median OWWS of four surgeons from gynecology was 46 and the median of the two surgeons from urology was 38.

**Fig. 2:**
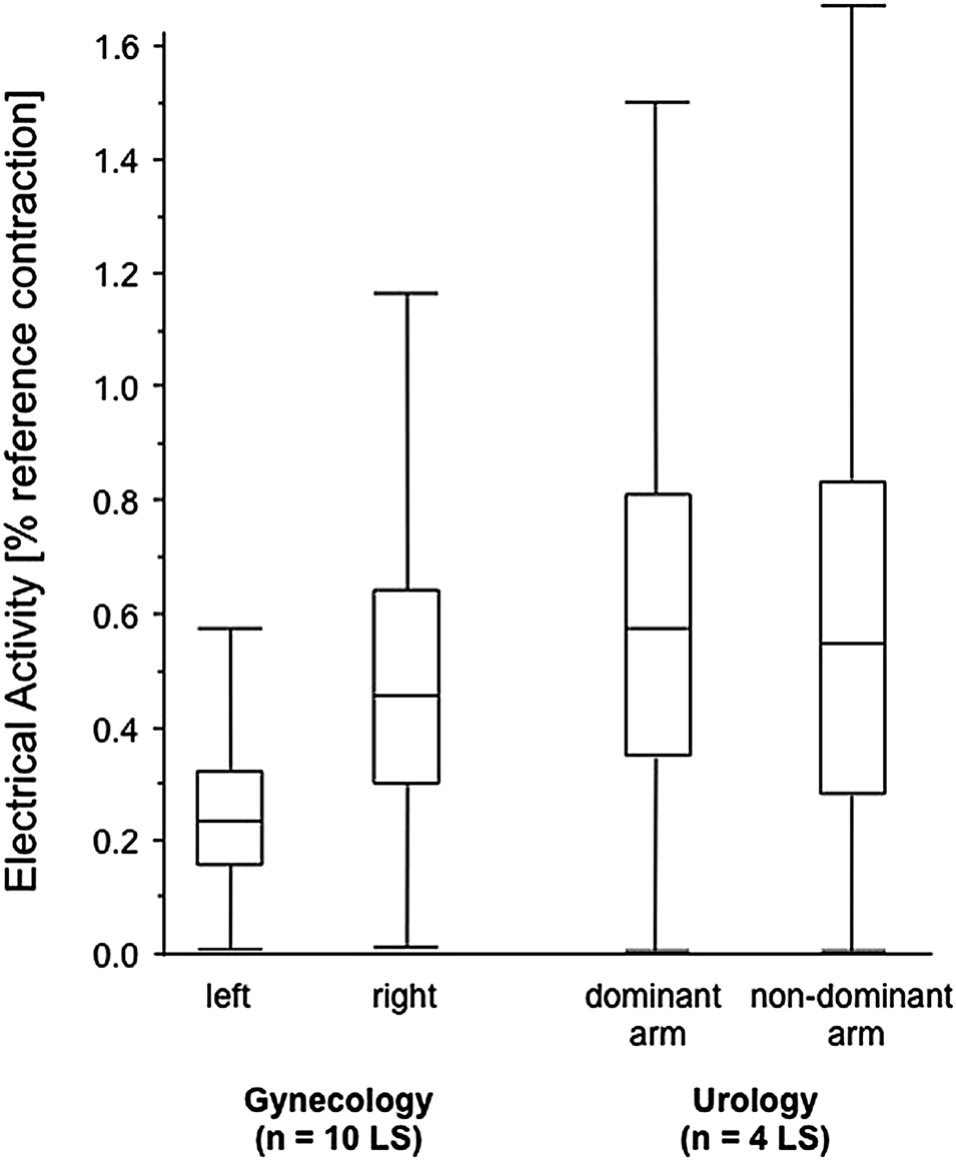
Electrical activity of the trapezius muscle during laparoscopic surgery in gynecology and urology. The electrical activity is given as the percent of the reference contraction performed with a 90° ante-version of both straight arms holding a 2-kg dumbbell in each hand

The trapezius muscle showed significant differences in muscle activity in gynecology surgery with higher activity in the right trapezius muscle. No differences between the left and right shoulder muscle activity were found in urology. Our observation also revealed that the surgeons of the gynecological surgical unit had to operate predominantly with their right arm irrespective of handedness. Surgeons in urology appeared free to use their dominant arm to conduct the operation. However, trapezius activity was elevated in both sides of the body (Fig. 2). The categorization of  right vs left hand and dominant vs non-dominant trapezius muscle, respectively, is accounted for in the different gynecology and urology settings.

**Fig. 3:**
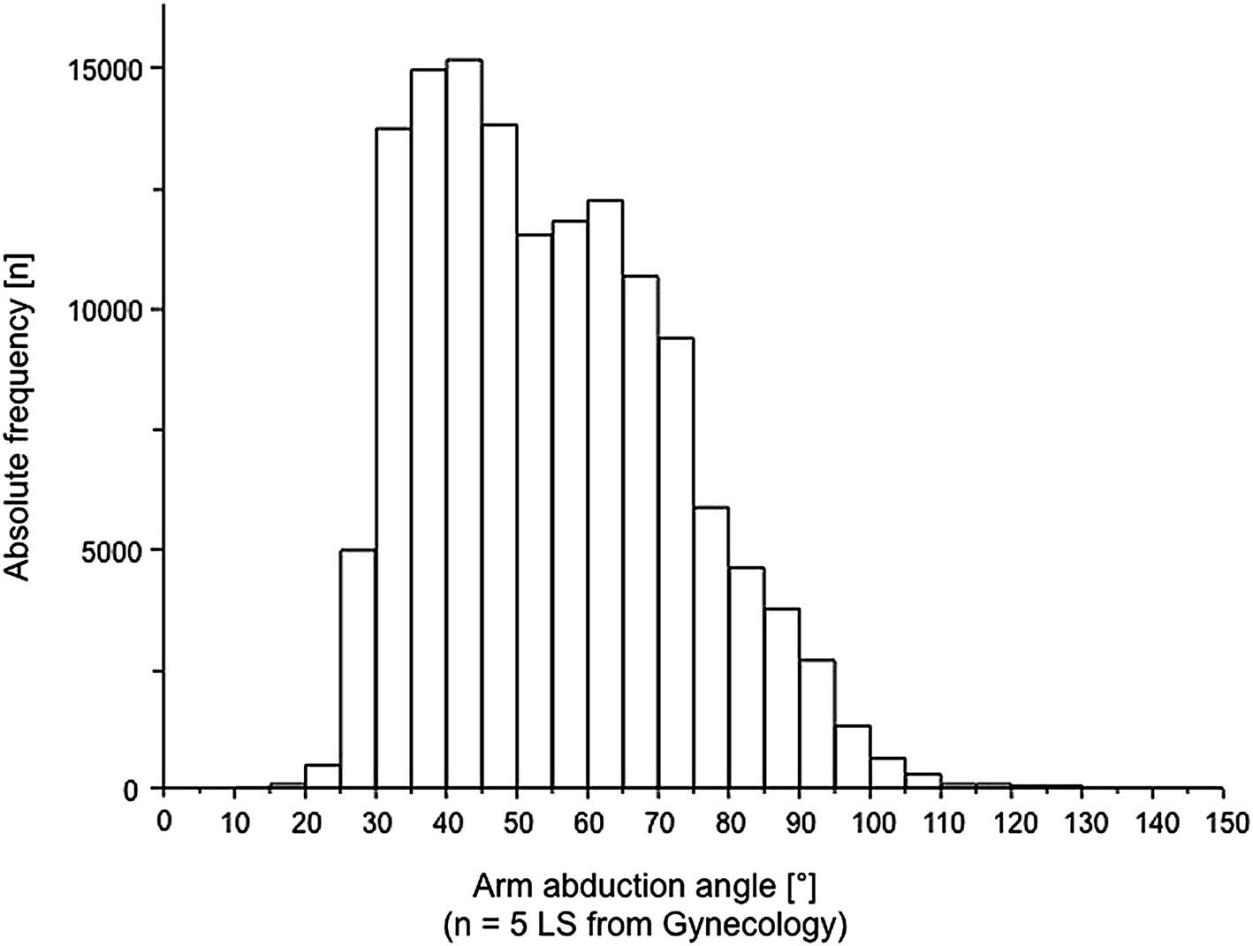
Frequency distribution of the arm abduction angle in gynecological laparoscopic surgery

Posture sensor of the right upper arm in gynecology showed a high amount of arm abduction during LS with a median of 52° and a range from 14° to 147° (Fig. 3).

The median heart rate was 85 (range 53–134) and did not change.

**Fig. 4:**
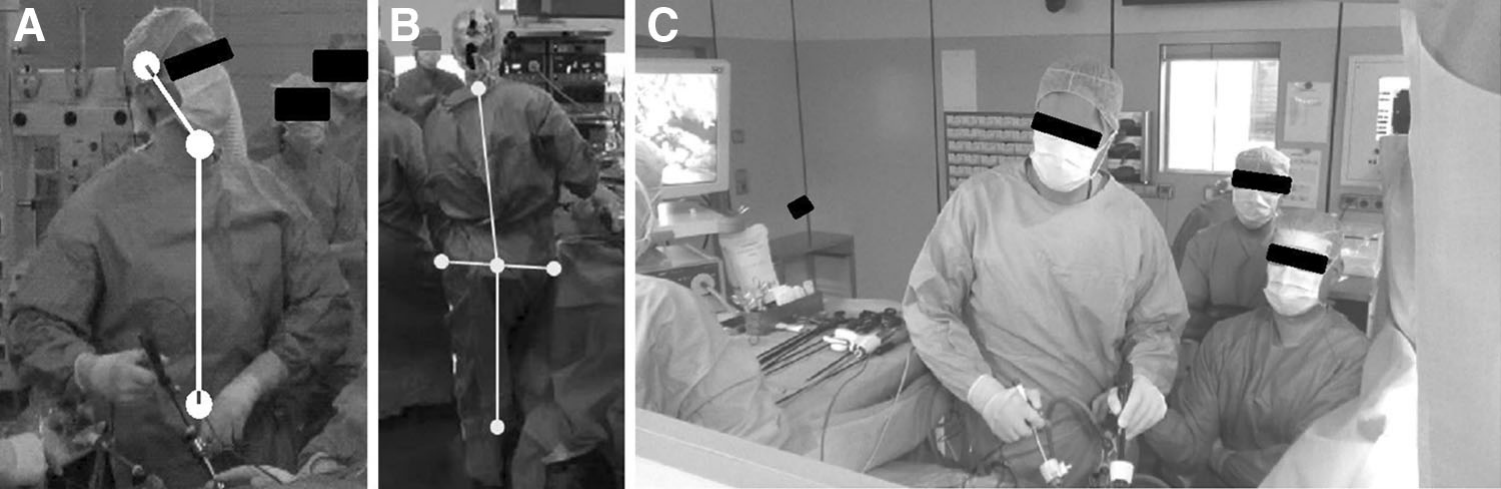
Examples of representative body posture during laparoscopic surgery

A 2D video analysis provided setting-related, task-related, and individual factors influencing surgeons’ postures. Settings with the main screen positioned above eye level caused surgeons to tilt their head backward (Fig. 4A). Task-related changes forced surgeons to flex and rotate their body to keep the screen in sight (Fig. 4B). Surgeons also showed individual postures despite similar settings, tasks, and individual ways to support their posture, such as leaning against the operating table or resting their arms on the table (Fig. 4C). Further prolonged elevated arm postures were identified indicating static muscle strain in the shoulder–neck region.

### Step 2: generate ideas for development of a support system

**Fig. 5:**
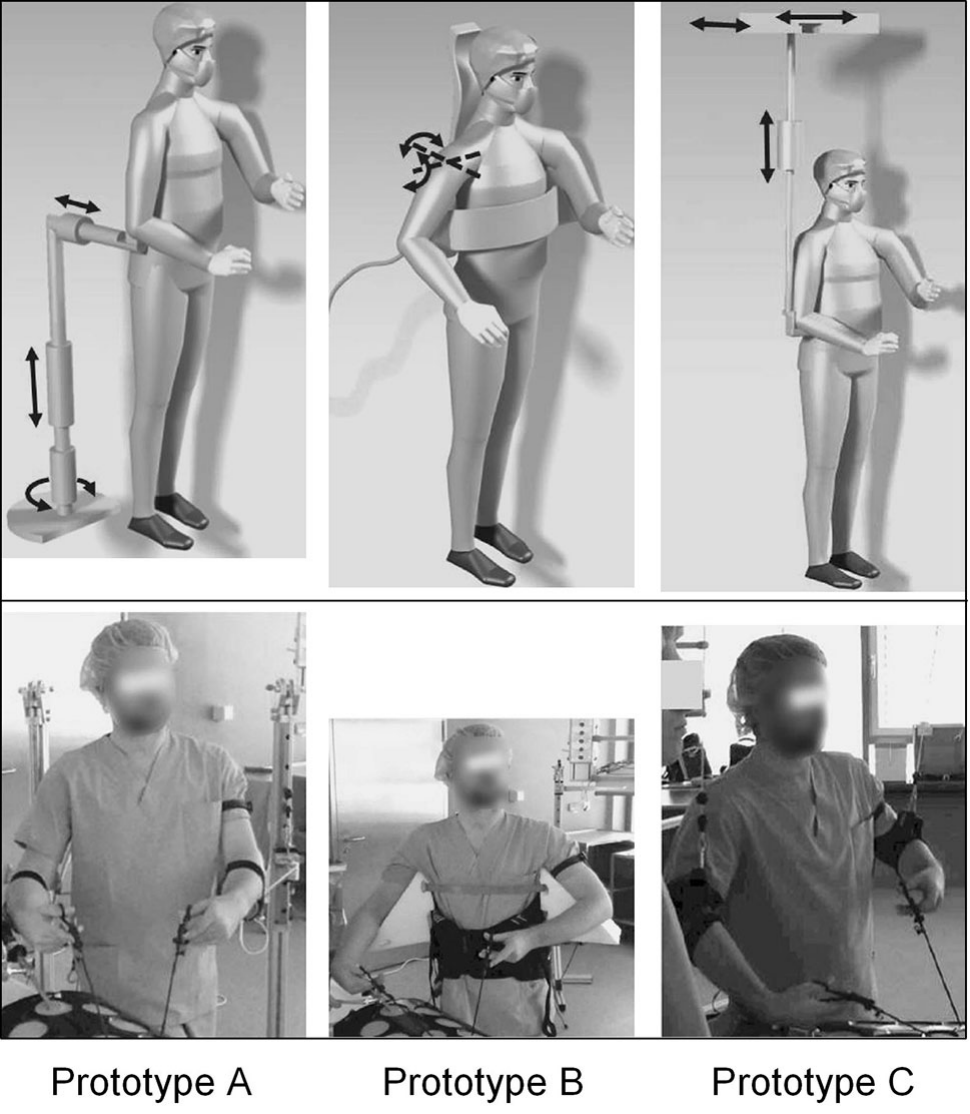
Prototypes of three different concepts of a support system

The results of step 1 confirmed the findings reported in the literature [4, 16, 17] regarding increased musculoskeletal pain and biomechanical stress in the shoulder–neck region. Furthermore several other aspects for ergonomic improvement during LS were observed such as the unergonomic position and placement of the monitor. However, recommendations for enhanced ergonomics monitor positioning were found in the literature [18]. Technical tools to reduce biomechanical stress in the shoulder–neck region are rare and may provide an opportunity to reduce physical stress that cannot be addressed by ergonomic monitor and table positioning only. Three basic concepts for the ASsyst were generated during the expert workshop. Concept A was a support for the entire lower arm that could either be used as a standalone device or construction fixed to the operating table. The second concept (concept B) was a pneumatic vest worn under the surgical work clothes. This vest could include air cushions that inflate on demand and support the upper arm of the surgeon. Concept C was a cable fixed to the ceiling that allows the surgeon to put their lower arm into a noose-like halter. These concepts resulted in three prototypes (Fig. 5).

### Step 3: test and evaluate elementary concepts/prototypes

**Fig. 6:**
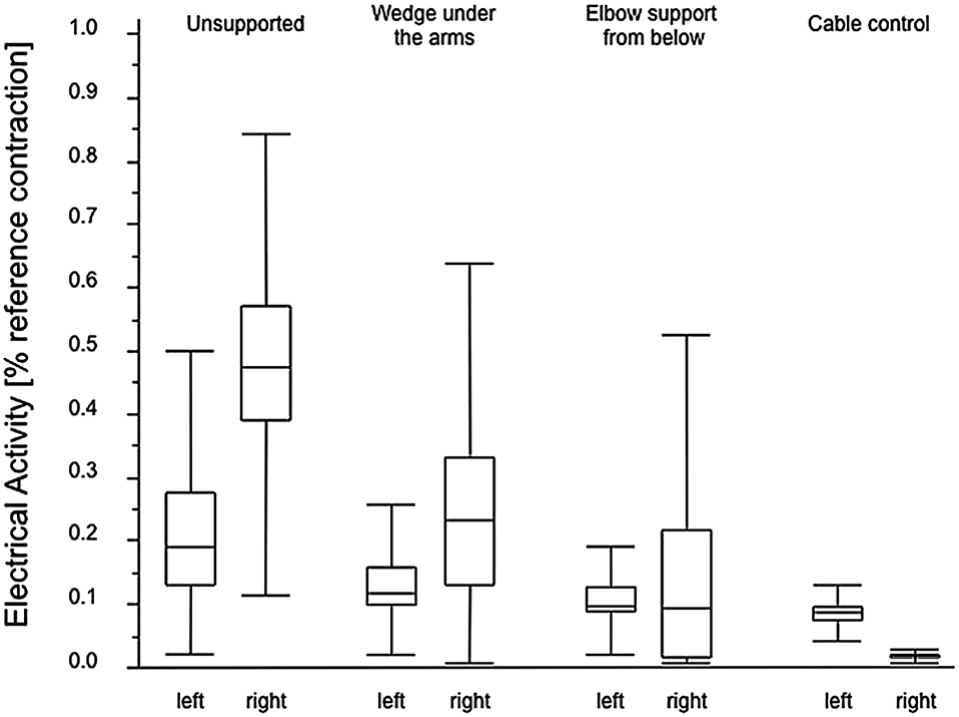
Electrical activity of the trapezius muscle performing the simulated laparoscopic surgery with and without prototypes of different supporting systems (simulated laparoscopic surgery was performed predominantly with the right arm). The electrical activity is given as a percent of the reference contraction which is performed with a 90° ante-version of both straight arms holding a 2-kg dumbbell in each hand

When using each of the three prototypes, the results demonstrated lower trapezius muscle activity compared to the unsupported simulation exercise (Fig. 6). Trapezius activity during the unsupported exercise performance was comparable to the activity recorded during step 1 under actual clinical conditions. The posture sensor placed at the upper arm confirmed that the arm abduction angle was comparable between all trials and prototypes.

### Step 4 and 5: development and evaluation of the prototype

Currently in progress.

## Discussion

LS is an established procedure in gynecology and urology with several limitations in workplace ergonomy. The research presented addresses ergonomic working conditions using a multi-modal concept for assessing the physicians’ physical stress and strain during LS. Based on this analysis, an ASsyst was designed and evaluated. The interdisciplinary approach of this project unites work physiologists and ergonomists, construction engineers, manufacturers and potential users of the ASsyst, i.e., gynecologic or urologic surgeons. The investigation of typical live LS performed by a variety of experienced surgeons ensures high external validation of the evaluation measures that were performed during step 1. Similarly, the evaluation of the three prototypes, based on simulated surgical laparoscopic procedures, led to high internal validity during step 3. The methods applied, e.g., SEMG, arm abduction angle, heart rate analysis, questionnaires, and 2D video analysis are well known from other studies [3, 9, 11, 19, 20], allowing a direct a comparison of the results.

The results of step 1 of this research project confirmed the demand for ergonomic optimization in LS, which was previously reported [3, 19, 21]. Our results revealed supplementary information that characterized physical strains in the shoulder–neck region. There were increased and asymmetric levels of trapezius muscle activity, a substantial amount of arm abduction angle, and stressful head positions during the laparoscopic interventions. Further, the mean OWWS determined from the NASA TLX questionnaire was 38 (urology) and 46 (gynecology), which seems to be in the normal range for laparoscopic procedures [11, 22]. Lee et al. [11] found that robotic-assisted laparoscopy decreased the OWWS through a change in the surgeons’ body posture. However, trapezius activity was not influenced using robotic-assisted surgery. They also reported that surgeons without experience in robotic-assisted surgery even showed increased levels of trapezius muscle activity when performing robotic-assisted surgery, [11] which demonstrates that the setting for the robotic surgery is not yet optimal for the surgeon.

Our results, combined with findings in the literature, underline the importance of ergonomic tools for LS specifically designed to reduce physical strains in the surgeon’s shoulder–neck region. In this context, a study on medical students indicated that a reduction of physical strain based on a subjective rating resulted in fewer errors during the performance of a laparoscopic task [23]. The concept of an ASsyst seems to address the strains in this specific body region. The experimental testing of three ASsyst prototypes during step 3 of this research project demonstrated a reduction in trapezius muscle activity. Although all prototypes decreased trapezius activity, the concept of an ASsyst that functions from below the elbow was favored based on the surgeons’ preference. With regard to its acceptance and practicability in a realistic operating room setting, this approach seemed to be the most promising.

The findings of Jafri et al. in 2012 [24] indicated lower energy consumption and fewer medical errors during simulated minimal access surgery when the surgeon’s arm was supported. It can be hypothesized that this ASsyst might be able to prevent surgeons from musculoskeletal injury in the shoulder–neck region and may also have a further benefit with regard to laparoscopic quality by reducing the medical errors caused by physical fatigue. These hypotheses will be further evaluated during step 4 and 5 of this research project.

Another added benefit that should be mentioned is that an ASsyst can easily be removed if the immediate conversion to standard LS is necessary, whether it is required in the event of an emergency or if the surgeon requires a different position. However, it has been reported that the conversion to standard LS due to medical emergency is very rare [25]. A review of the literature shows only few cases of malfunction of a well-known robotic-assisted laparoscopic system, the da Vinci system [26]. The robotic-assisted laparoscopic system has yet to be ergonomically evaluated and may lead to high physical strain in surgeons as well [11]. Further, there may even be an advantage of an ASsyst in comparison to robotic-assisted laparoscopy such as the da Vinci system, with regard to the reduction of physical stress and strain as well as patients’ safety. Comparative data on physical strain with and without use of the ASsyst will be available after the completion of step 4 and 5.

## Conclusions

It has been demonstrated that there is a need for ergonomic optimization during LS. As the shoulder–neck region of the surgeon is exposed to major physical stress as well as other physical locations, an ASsyst is likely to improve ergonomics during LS. We were able to demonstrate that a specifically designed device can be used to supplement conventional laparoscopic settings and provide ergonomic advantages which reduce measurable stress and strain for the surgeon.
